# Epigenetic alterations of the keratin 13 gene in oral squamous cell carcinoma

**DOI:** 10.1186/1471-2407-14-988

**Published:** 2014-12-20

**Authors:** Kaori Naganuma, Mitsutoki Hatta, Tetsuro Ikebe, Jun Yamazaki

**Affiliations:** Department of Physiological Science and Molecular Biology, Fukuoka Dental College, Fukuoka, 814-0193 Japan; Department of Oral and Maxillofacial Surgery, Fukuoka Dental College, Fukuoka, 814-0193 Japan

**Keywords:** Keratin 13 (KRT13), Oral squamous cell carcinoma (OSCC), Polycomb repressive complex 2 (PRC2), Gene silencing

## Abstract

**Background:**

Epigenetic modifications play important roles in the regulation of gene expression determining cellular phenotype as well as various pathologies such as cancer. Although the loss of keratin 13 (KRT13) is reportedly linked to malignant transformation of oral epithelial cells, the molecular mechanisms through which KRT13 is repressed in oral squamous cell carcinoma (OSCC) remain unclear. The aim of this study is to identify the epigenetic alterations of the KRT13 gene in OSCCs.

**Methods:**

We investigated KRT13 expression levels and chromatin modifications of the KRT13 promoter in the three OSCC cell lines (HSC4, HSC3, and SAS). The expression levels of KRT13 protein and mRNA were analyzed by western blotting and quantitative reverse-transcription polymerase chain reaction, respectively, and the localization of KRT13 protein was detected by immunofluorescence. DNA methylation and histone modifications in the KRT13 promoter were determined by bisulfite sequencing and chromatin immunoprecipitation (ChIP), respectively. For the pharmacological depletion of Polycomb repressive complex 2 (PRC2), cells were treated with 3-deazaneplanocin A (DZNep).

**Results:**

KRT13 expression was transcriptionally silenced in the HSC3 and SAS cells and post-transcriptionally repressed in the HSC4 cells, while the KRT13 promoter was hypermethylated in all of the three OSCC cell lines. ChIP analysis revealed that PRC2-mediated trimethylation of Lys 27 on histone H3 (H3K27me3) was increased in the KRT13 promoter in the HSC3 and SAS cells. Finally, we demonstrated that the treatment of SAS cells with DZNep reactivated the transcription of KRT13 gene.

**Conclusions:**

Our data provide mechanistic insights into the epigenetic silencing of KRT13 genes in OSCC cells and might be useful for the development of diagnostic markers and novel therapeutic approaches against OSCCs.

**Electronic supplementary material:**

The online version of this article (doi:10.1186/1471-2407-14-988) contains supplementary material, which is available to authorized users.

## Background

Epigenetic mechanisms play important roles in the regulation of gene expression and phenotypic plasticity. The addition of a methyl group to the cytosine of a CpG dinucleotide (i.e., DNA methylation) in the promoter region of genes commonly mediates gene repression and acts as a silencing mechanism [1]. Post-translational modifications of histone tails are important regulatory markers for generating transcriptionally active and inactive chromatin. For instance, the trimethylation of Lys 4 on histone H3 (H3K4me3) is associated with gene activation, while the methylation of H3K27 (H3K27me3) and H3K9 (H3K9me2 and H3K9me3) is often connected to gene repression [2, 3]. These epigenetic modifications dynamically regulate the chromatin architecture of promoter regions leading to the establishment of gene expression patterns. Polycomb repressive complex 2 (PRC2) comprises four core components (Ezh2, Suz12, Eed, and RbAp46/48) and several other proteins [4]. Ezh2 contains histone methyltransfease activity and plays an important role in the methylation of H3K27 mediated by PRC2. Dysregulation of PRC2 has been linked to several human cancers including lymphoma, squamous cell carcinoma, and breast and prostate cancer [5–9].

Oral squamous cell carcinoma (OSCC) is the most common neoplasm of the oral cavity and has poor clinical outcomes associated with recurrence and metastasis [10]. The Keratin 13 (KRT13) gene encodes a type I acidic keratin which is expressed in the differentiated cells of non-cornified stratified squamous epithelia [11–13]. Notably, the disappearance of KRT13 is often seen in OSCC lesions, while KRT13 is expressed in normal non-cornified oral mucosa [14–19]. In addition, KRT13-negative OSCC is associated with a high potential for local recurrence [20]. Although the loss of KRT13 is correlated with the cellular transformation of oral epithelial cells, the epigenetic mechanisms by which KRT13 is repressed in OSCCs remain unclear.

In this study, we examined the epigenetic alterations in OSCC cells by focusing on the silencing mechanisms of the KRT13 gene and showed elevated KRT13 promoter DNA methylation and repressive histone modifications in OSCC cell lines. Furthermore, we found a PRC2 inhibitor effective for restoring KRT13 transcription. Our findings provide molecular insights into the epigenetic silencing of the KRT13 gene in OSCC cells as well as important implications for the development of diagnostic markers and novel therapeutic approaches.

## Methods

### Ethics statement

All experiments in this manuscript have been approved by the Fukuoka Dental College Institutional Biosafety Committee.

### Cells and drug treatment

HSC3 and HSC4 cells were cultured as described previously [21]. Immortalized human keratinocyte HaCaT cells and OSCC-derived SAS cells were maintained in Dulbecco’s Modified Eagle’s Medium supplemented with 10% fetal bovine serum, 50 units/ml penicillin, and 50 μg/ml streptomycin and maintained at 37°C with 5% CO_2_. The potent PRC2 inhibitor 3-deazaneplanocin A (DZNep) was purchased from Sigma-Aldrich (St. Louis, MO). Cells were seeded the day before the drug treatment, and DZNep (10 μM) was added to the culture medium for 24 h or 72 h.

### DNA methylation analysis

Genomic DNA was extracted from cells using the NucleoSpin Tissue kit (Macherey-Nagel, Düren, Germany) and subjected to bisulfite modification using the MethylEasy Xceed kit (Human Genetic Signatures, Randwick, Australia) according to the manufacturer’s instructions. The primers used for amplification of the KRT13 promoter after bisulfite modification were 5′-TGGAGTAGATGAAGTGCTAAGAAGG-3′ and 5′-AACAAAAAGAATGATTCAGAGGGG-3′. Polymerase chain reaction (PCR) was performed with EX-taq DNA polymerase (Takara Bio Inc., Shiga, Japan), followed by TA cloning into a pMD20-T vector (Takara Bio Inc.) and sequencing of individual clones.

### Quantitative RT-PCR

Total RNA was extracted from cells using the NucleoSpin RNA kit (Macherey-Nagel) and reverse transcription was performed using a PrimeScript RT reagent kit (Takara Bio Inc.) according to the manufacturer’s instructions. Quantitative reverse-transcription PCR (RT-PCR) was performed on an ABI 7500 real-time PCR system (Applied Biosystems, Carlsbad, CA) using SYBR Premix Ex Taq II (Takara Bio Inc.). The primers used for KRT13 were 5′-GACCGCCACCATTGAAAACAA-3′ and 5′-TCCAGGTCATTAGACAGAG-3′. GAPDH was used as a reference gene for normalization. The primers used for GAPDH were 5′-GGAGCGAGATCCCTCCAAAAT-3′ and 5′-GGCTGTTGTCATACTTCTCATGG-3′. PCR conditions were as follows: 95°C for 30 sec, followed by 45 cycles of 95°C for 10 sec and 60°C for 1 min. The relative standard curve method was used to quantify relative mRNA levels of KRT13 and GAPDH.

### Western blotting

Cells were scraped and resuspended in RIPA buffer (25 mM Tris–HCl pH 7.4, 150 mM NaCl, 0.1% SDS, 1% Noidet P-40, 5 mM EDTA, 1% sodium deoxycholate). Total protein extracts (5–10 μg) were resolved by electrophoresis on 4–20% polyacrylamide-SDS gels and transferred onto polyvinylidene fluoride membranes. The membranes were blocked with 4% (w/v) ECL Prime blocking agent (GE Healthcare Life Sciences, Backinghamshire, UK) in 0.1% Tween-TBS and probed with primary antibodies, followed by horseradish peroxidase-conjugated secondary antibodies. The specific antigen-antibody interactions were detected on a LAS-2000 imaging system (Fuji Film, Tokyo, Japan) using an ECL Prime Western Blotting Detection Reagent (GE Healthcare Life Sciences). Quantification of band intensity was performed using ImageJ 1.47v (National Institute of Health, Bethesda, MD). Relative expression levels were normalized to β-actin or histone H3. The following primary antibodies were used in this study: anti-keratin 13 (EPR3671; Abcam, Cambridge, UK; dilution, 1:1000), anti-Ezh2 (#5246; Cell Signaling Technology, Danvers, MA; dilution, 1:1000), anti-Suz12 (#3737; Cell Signaling Technology; dilution, 1:1000), anti-histone H3 (#4499; Cell Signaling Technology; dilution, 1:1000), anti-trimethyl histone H3 (Lys27) (MAB323B; MAB Institute Inc., Sapporo, Japan; dilution, 1:1000), and anti-β-actin (sc-47778; Santa Cruz Biotechnology, Dallas, TX; dilution, 1:2000).

### Immunofluorescence

Cells were plated on Nunc Lab-Tek chamber slides (177429; Thermo Scientific, Waltham, MA), incubated for 24 h, fixed with 4% paraformaldehyde in PBS for 20 min at 21–25°C, permeabilized with 0.1% Triton X-100 for 30 min, and washed three times with PBS. Cells were blocked with 1% bovine serum albumin and 0.1% Tween 20 in PBS and probed with anti-keratin 13 (EPR3671; Abcam, dilution; 1:100), followed by anti-rabbit immunoglobulin-G (IgG) antibody conjugated with Alexa Fluor 488 (A-11034; Life Technologies, Carlsbad, CA; dilution, 1:800). Nuclei were counterstained with DAPI (P36935; Life Technologies). Fluorescence imaging was performed and images were captured using a Biorevo microscope (BZ-9000; Keyence, Osaka, Japan).

### Chromatin immunoprecipitation

Cells were fixed in culture medium containing 1% formaldehyde for 10 min at 21–25°C and incubated in NP-40 buffer (10 mM Tris–HCl pH 8.0, 10 mM NaCl, 0.5% NP-40) for 5 min at 21–25°C. Cell were harvested and resuspended in SDS lysis buffer (50 mM Tris–HCl pH 8.0, 1% SDS, 10 mM EDTA), followed by 5-fold dilution in chromatin immunoprecipitation (ChIP) dilution buffer (50 mM Tris–HCl pH 8.0, 167 mM NaCl, 1.1% Triton X-100, 0.11% sodium deoxycholate). Chromatin was sonicated using a Bioruptor (Cosmo Bio Co., Tokyo, Japan) at medium power ten times for 20 sec. Ten micrograms of soluble sheared chromatin was incubated overnight at 4°C with protein G magnetic beads (#9006; Cell Signaling Technology) bound to 2 μg anti-histone H3 (#4620; Cell Signaling Technology), anti-trimethyl histone H3 (Lys4) (#9751; Cell Signaling Technology), anti-trimethyl histone H3 (Lys27) (#9733; Cell Signaling Technology), or control IgG (Cell Signaling Technology #2729), followed by sequential washing with low salt RIPA buffer (50 mM Tris–HCl pH 8.0, 150 mM NaCl, 1 mM EDTA, 0.1% SDS, 1% Triton X-100, 0.1% sodium deoxycholate), high salt RIPA buffer (50 mM Tris–HCl pH 8.0, 500 mM NaCl, 1 mM EDTA, 0.1% SDS, 1% Triton X-100, 0.1% sodium deoxycholate), wash buffer (50 mM Hepes-KOH pH 7.5, 500 mM LiCl, 1 mM EDTA, 1% NP-40, 0.7% sodium deoxycholate), and TE buffer. Immune complexes were then eluted by incubation for 20 min at 65°C with ChIP direct elution buffer (50 mM Tris–HCl, pH 8.0, 10 mM EDTA, 1% SDS), and the cross-linking was reversed by incubating overnight at 65°C. DNA was purified using the GenElute PCR Clean-up kit (Sigma-Aldrich) and subjected to quantitative PCR on an ABI 7500 real-time PCR system (Applied Biosystems). The primers used for the KRT13 promoter were 5′-TTGTGGGAAACAGAAGTGTAGTTGGC-3′ and 5′-GGTGAGAGCAGGATTGAGAGCAGGT -3′.

### Statistics

All values are presented as the means ± SEM for each group. Statistical analysis was performed using Student’s *t*-test to compare the means of two groups or by a one-way analysis of variance followed by Dunnett’s post hoc test for more than three groups. p < 0.05 was considered significant.

## Results

### KRT13 repression by multiple mechanisms in OSCC cells

**Figure 1:**
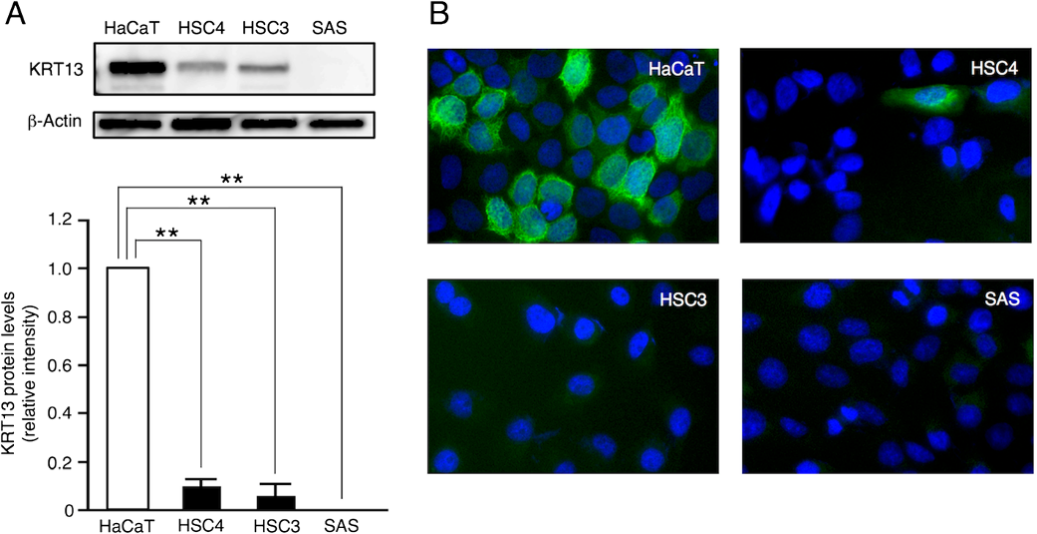
**Loss of KRT13 protein in oral squamous cell carcinoma (OSCC) cells. (A)** KRT13 protein levels in three OSCC cell lines and HaCaT cells were examined by western blotting. Representative images are shown. Fold change in KRT13 protein was calculated relative to band intensity of the HaCaT cells and normalized to that of β-actin. The means ± SEM for each group (n = 4–5) are shown. Statistical analysis was performed by a one-way analysis of variance followed by Dunnett’s post hoc test. **p < 0.01. **(B)** Expression of KRT13 (green) was analyzed by immunofluorescence staining, and nuclei were visualized with DAPI staining (blue).

**Figure 2:**
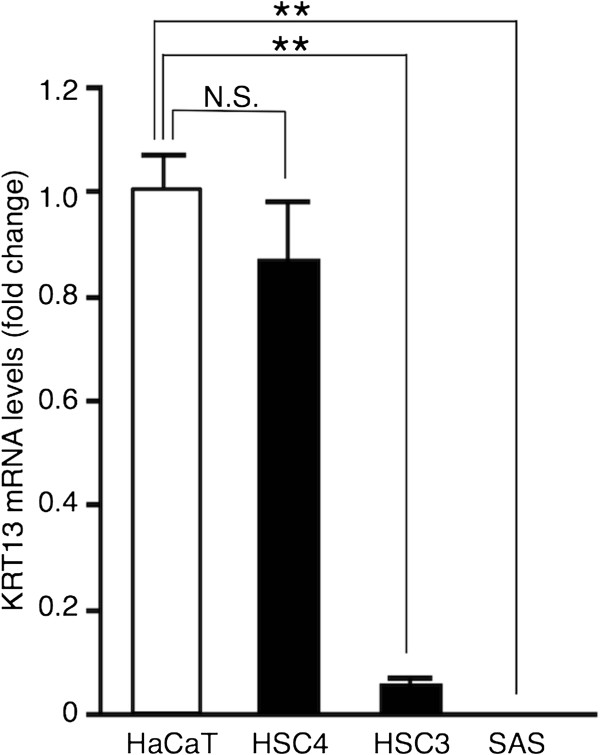
**Silencing of the KRT13 gene in poorly differentiated OSCC cells.** KRT13 mRNA levels in three OSCC cell lines and HaCaT cells were analyzed by quantitative reverse-transcription polymerase chain reaction (RT-PCR). Fold change in KRT13 mRNA was normalized to GAPDH and calculated relative to that of the HaCaT cells. The means ± SEM for each group (n = 3) are shown. Statistical analysis was performed by a one-way analysis of variance followed by Dunnett’s post hoc test. **p < 0.01, N.S. (not significant).

First, we examined KRT13 expression levels in the differentiated OSCC cell line (HSC4), in the poorly differentiated OSCC cell lines (HSC3 and SAS), and in the immortalized human keratinocyte HaCaT cell line [22–24]. Western blotting showed that KRT13 protein levels were significantly decreased in the HSC4 and HSC3 cells compared with the HaCaT cells, and almost absent in the SAS cells (Figure 1A). Immunofluorescence microscopy revealed high cytoplasmic expression of KRT13 protein in the HaCaT cells, but not in the OSCC cell lines (Figure 1B and Additional file 1: Figure S1). To investigate whether the reduction in KRT13 protein levels was due to the reduced expression of KRT13 mRNA, we performed quantitative RT-PCR analysis. As shown in Figure 2, KRT13 mRNA levels were significantly decreased in the HSC3 and SAS cells, but not in the HSC4 cells, compared with HaCaT cells. These results suggest that KRT13 expression is likely to be repressed by translational inhibition or protein degradation in the HSC4 cells and transcriptionally silenced in the HSC3 and SAS cells.

### KRT13 promoter methylated in OSCC cells

**Figure 3:**
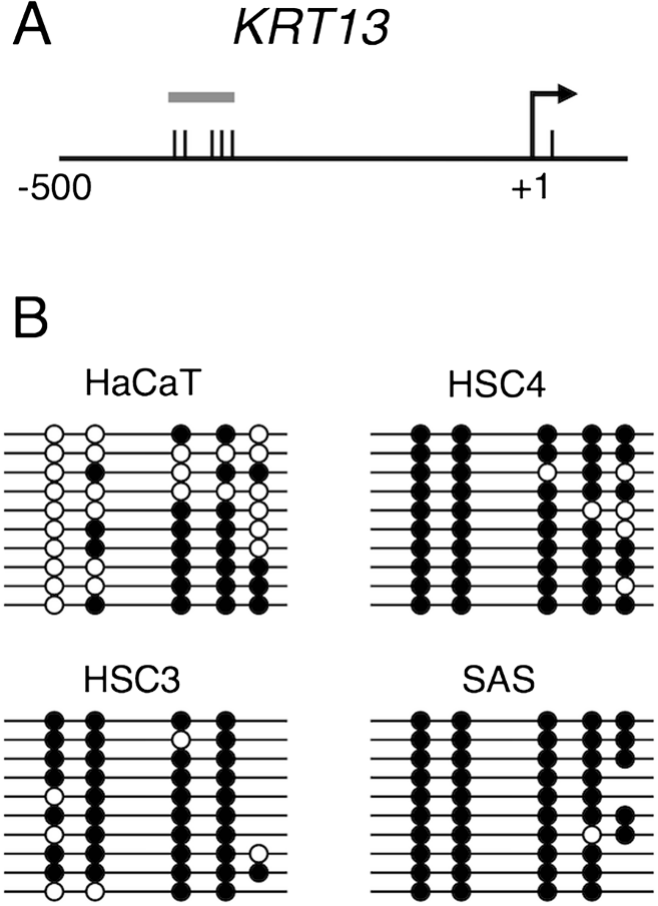
**DNA methylation status of the KRT13 promoter in OSCC cells. (A)** Schematic of the KRT13 gene promoter region. The tick represents individual CpG sites and the arrow represents the transcription start site. The bar indicates the region (-470 to -190) subjected to DNA methylation analysis. **(B)** CpG methylation status of the KRT13 promoter in the three OSCC cell lines and HaCaT cells. Open and closed circles represent methylated and unmethylated CpG sites, respectively. The absence of circles indicates missing data.

Since it has been reported that the methylation status of the promoter region is generally linked to gene repression [1], we examined the DNA methylation status of the KRT13 promoter in the OSCC cells. The CpG sites within the proximal promoter region (-470 to -190) were analyzed by bisulfite sequencing (Figure 3A). As shown in Figure 3B, the KRT13 promoter was partially methylated (<50%) in the HaCaT cells and highly methylated (≥90%) in the three OSCC cell lines (HSC4, HSC3, and SAS). These data show that the KRT13 promoter was aberrantly hypermethylated in all of the examined OSCC cell lines.

### Alterations of histone H3 methylation patterns in the KRT13 promoter in OSCC cells

**Figure 4:**
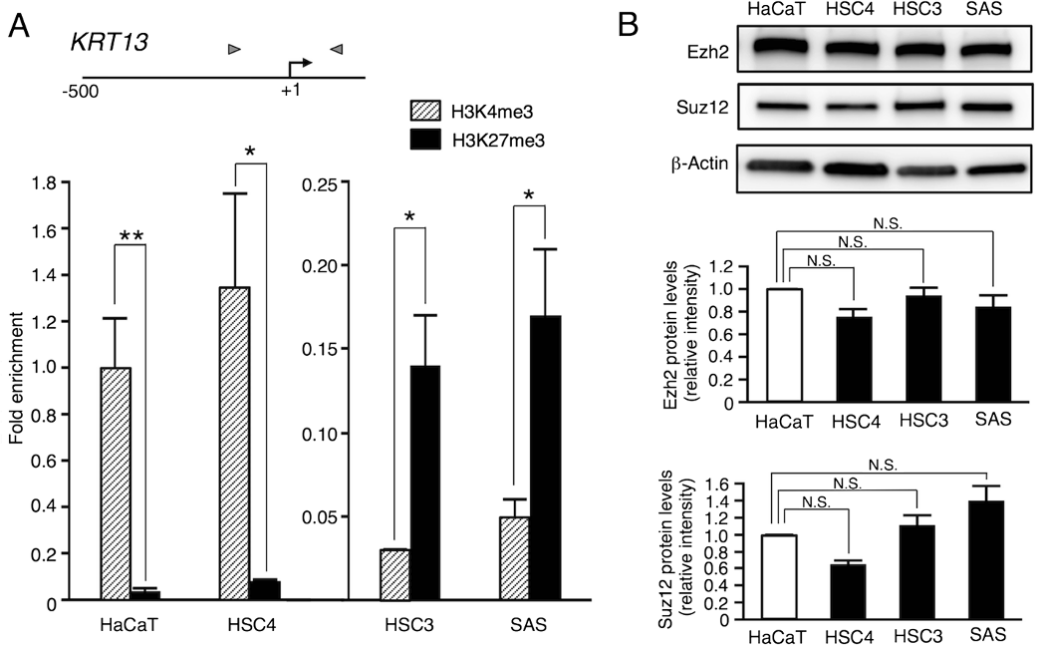
**Alterations of histone modifications in the KRT13 promoter in OSCC cells. (A)** Schematic of the promoter region of the KRT13 gene. The arrow represents the transcription start site, and arrowheads indicate the primer set used for quantitative PCR. The methylation patterns of histone H3 (H3K4me3 and H3K27me3) in the KRT13 promoter in the three OSCC cell lines and HaCaT cells were examined by chromatin immunoprecipitation (ChIP) assay. Fold enrichment of histone H3 modifications was normalized to total histone H3 and calculated relative to that of HaCaT cells. The value of H3K4me3 in the HaCaT cells was set to 1. The means ± SEM for each group (n = 3–5) are shown. Statistical analysis was performed using Student’s *t*-test. *p < 0.05, **p < 0.01. **(B)** The levels of PRC2 proteins (Ezh2 and Suz12) were analyzed in the three OSCC cell lines and HaCaT cells by western blotting. Representative images are shown. Fold change in Ezh2 and Suz12 proteins were normalized to β-actin and calculated relative to band intensity of the HaCaT cells. The means ± SEM for each group (n = 3) are shown. Statistical analysis was performed by a one-way analysis of variance followed by Dunnett’s post hoc test. N.S. (not significant).

DNA methylation of the KRT13 promoter did not correlate with KRT13 gene repression. Thus, it is likely that alterations of histone modifications are responsible for KRT13 gene repression in OSCC cells. It has been reported that H3K4me3 is associated with active transcription, while H3K27me3 is generally linked to gene repression [2, 3]. We therefore examined histone H3 methylation patterns at the KRT13 promoter in the HaCaT cells and the three OSCC cell lines. ChIP analysis demonstrated that high levels of H3K4me3 but low levels of K27me3 were detected in the HaCaT and HSC4 cells, in which KRT13 mRNA was highly expressed. On the other hand, HSC3 and SAS cells showed a histone code switch to high H3K27me3 but low K4me3 (Figure 4A). Additionally, we observed high levels of H3K9me2 and K9me3, and low occupancy of RNA polymerase II (RNAP II) at the KRT13 promoter in the SAS cells compared with the HaCaT cells (Additional file 2: Figure S2A and B). Since PRC2 is known to play an important role in gene repression through the demethylation of H3K4 and the trimethylation of H3K27 and is linked to human cancer [5–9], we examined the expression levels of PRC2 component proteins (Ezh2 and Suz12) by western blotting. As shown in Figure 4B, we found no significant differences in the levels of Ezh2 and Suz12 proteins among the four cell lines. These data suggest that the histone H3 methylation patterns in the KRT13 promoter of the poorly differentiated OSCC cell lines (HSC3 and SAS) are distinct from those of the HaCaT and HSC4 cells and that the aberrant targeting of PRC2 to the KRT13 promoter may cause this site-specific alteration.

### Reactivation of KRT13 transcription by the PRC2 inhibitor DZNep in OSCC cells

**Figure 5:**
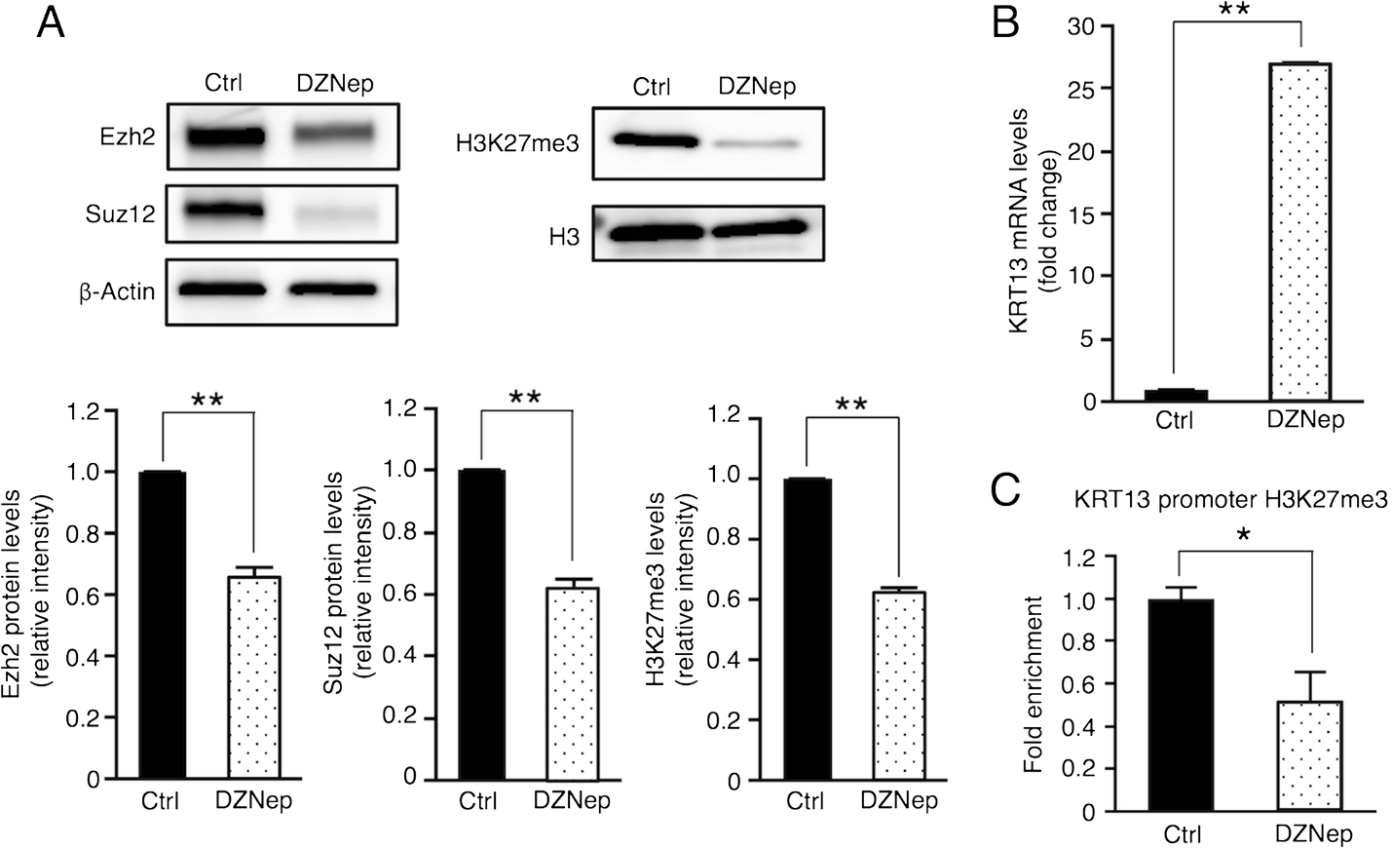
**Effects of 3-deazaneplanocin A (DZNep) on KRT13 expression in OSCC cells. (A)** SAS cells were treated with 10 μM DZNep for 24 h, and the levels of the PRC2 proteins (Ezh2 and Suz12) and H3K27me3 were examined by western blotting. Fold change in PRC2 proteins and H3K27me3 were normalized to β-actin and total histone H3, respectively, and calculated relative to band intensity of the control condition samples. The means ± SEM for each group (n = 3) are shown. Statistical analysis was performed using Student’s *t*-test. **p < 0.01. **(B)** KRT13 mRNA levels were analyzed by quantitative RT-PCR. Fold change in KRT13 mRNA was normalized to GAPDH and calculated relative to that of the cells under the control condition. The means ± SEM for each group (n = 3) are shown. Statistical analysis was performed using Student’s *t*-test. **p < 0.01. **(C)** H3K27me3 levels in the KRT13 promoter were analyzed by ChIP assay. Fold enrichment of histone H3K27me3 was normalized to that of total histone H3 and calculated relative to that of the cells under the control condition. The means ± SEM for each group (n = 3–5) are shown. Statistical analysis was performed using Student’s *t*-test. *p < 0.05.

**Figure 6:**
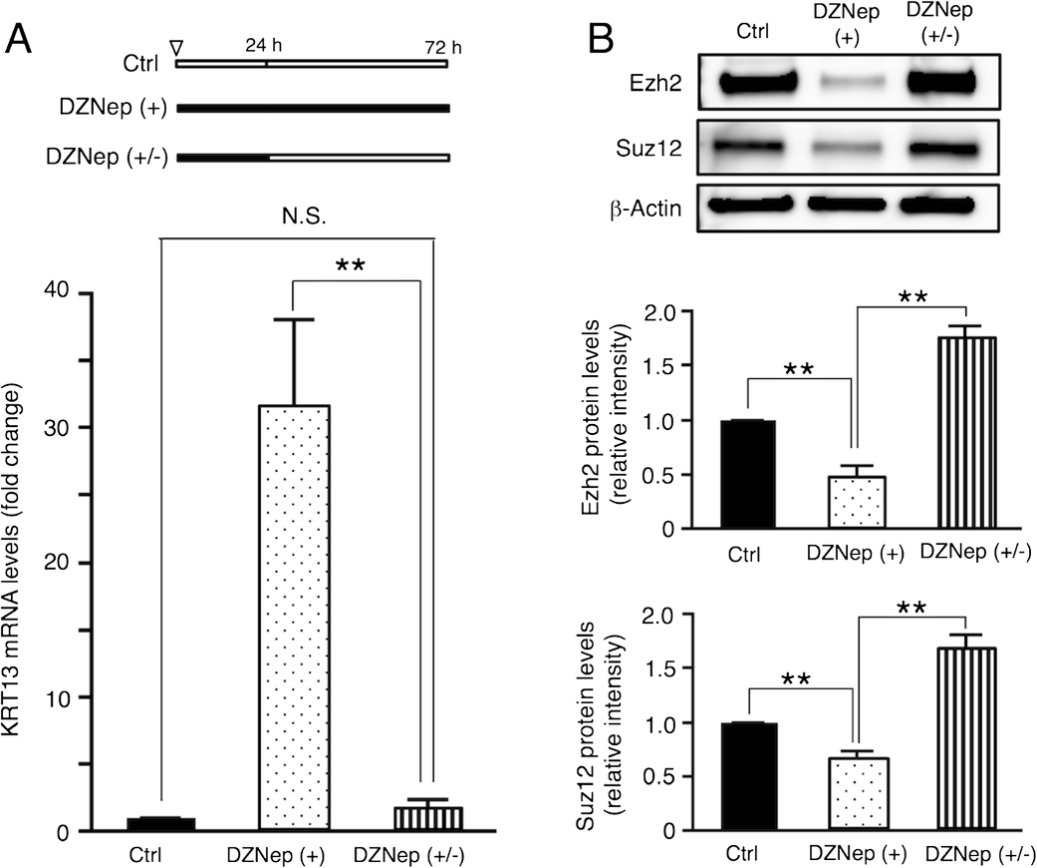
**Reversibility of DZNep-induced KRT13 expression in OSCC cells. (A)** Schematic of DZNep treatment time. The inverted triangle denotes the starting point. White and black boxes represent incubation without and with 10 μM DZNep, respectively. SAS cells were incubated without DZNep for 72 h (control), with DZNep for 72 h (DZNep (+)), or with DZNep for 24 h followed by the removal of DZNep (DZNep (+/-)). KRT13 mRNA levels were analyzed by quantitative RT-PCR. Fold change in KRT13 mRNA was normalized to GAPDH and calculated relative to that of the cells under the control condition. The means ± SEM for each group (n = 3) are shown. Statistical analysis was performed by a one-way analysis of variance followed by Dunnett’s post hoc test. **p < 0.01, N.S. (not significant). **(B)** Ezh2 and Suz12 protein levels were examined by western blotting. Representative images are shown. Fold change in Ezh2 and Suz12 proteins was normalized to β-actin and calculated relative to band intensity of the control condition samples. The means ± SEM for each group (n = 4) are shown. Statistical analysis was performed by a one-way analysis of variance followed by Dunnett’s post hoc test. **p < 0.01.

To determine whether PRC2-mediated transcriptional repression is responsible for KRT13 gene silencing, we examined the effects of the potent PCR2 inhibitor DZNep on KRT13 mRNA expression in the SAS cells. As shown by western blotting (Figure 5A), treatment of the SAS cells with 10 μM DZNep for 24 h decreased the Ezh2 and Suz12 protein levels and H3K27 trimethylation levels, and KRT13 mRNA expression was markedly increased by almost 30-fold after DZNep treatment (Figure 5B). In addition, the H3K27me3 levels in the KRT13 promoter were reduced in the SAS cells treated with DZNep (Figure 5C). We then investigated the reversibility of DZNep-induced KRT13 transcription in SAS cells, which had been treated with DZNep for 24 h. KRT13 mRNA levels were increased after additional exposure to DZNep for 48 h, whereas the mRNA levels were reversed for 48 h after withdrawing DZNep (Figure 6A). As shown in Figure 6B, the Ezh2 and Suz12 protein levels were decreased by DZNep treatment, and increased more than normal levels after drug removal. Taken together, our data suggest that DZNep induces the down-regulation of Ezh2 proteins and reactivates the transcription of KRT13 gene in SAS cells and that the effects of DZNep treatment on SAS cells are not heritable changes.

## Discussion

This study provides molecular insights into the epigenetic silencing of the KRT13 gene in OSCC cells. Hypermethylation of CpG island promoters is known to play a critical role in transcriptional silencing [1], and it has recently been reported that DNA methylation can directly silence genes with non-CpG island promoters [25]. We therefore investigated the DNA methylation status of the KRT13 promoter. According to the defining criteria of CpG islands [26], the KRT13 promoter is classified as a non-CpG promoter. Bisulfite sequencing revealed that the KRT13 promoter was hypermethylated in all of the transformed OSCC cells tested compared with immortalized keratinocytes (Figure 3). Interestingly, Marsit et al. [27] reported that the KRT13 gene was identified as one of the methylated genes associated with aggressive bladder cancer. It is possible that the aberrant DNA methylation patterns of the KRT13 promoter are a hallmark for certain types of cancer.

We evaluated KRT13 expression levels by western blotting, immunocytochemistry, and quantitative RT-PCR. KRT13 protein levels were significantly reduced in all three OSCC cell lines (Figure 1), while KRT13 transcription levels were decreased in the poorly differentiated OSCC cell lines (HSC3 and SAS), but not in the differentiated OSCC cell line HSC4 (Figure 2). This finding is consistent with a previous immunohistochemistry study using paraffin sections of oral carcinoma *in situ* [16]. Thus, it is likely that OSCC cells repress KRT13 through multiple mechanisms. Our bisulfite sequencing data suggest that DNA methylation in the KRT13 promoter does not directly mediate transcriptional silencing, and that additional events, such as histone modifications, are required for KRT13 gene silencing in the poorly differentiated OSCC cells.

Epigenetic changes of histone modifications (low H3K4me3 and high H3K27me3) in the KRT13 promoter were identified in the HSC3 and SAS cells (Figure 4). We focused on PRC2-mediated H3K27me3, since it has been reported that H3K27me3 and the PRC2 component Ezh2 are involved in squamous cell differentiation [28], and that deregulation of PRC2 proteins is closely related to several human tumors [5–9]. To examine the recruitment of PRC2 proteins to the KRT13 promoter in SAS cells, we pharmacologically depleted PRC2 proteins. DZNep was previously reported to deplete the cellular levels of PRC2 proteins and inhibit H3K27 trimethylation [29]. DZNep treatment reduced PRC2 proteins and H3K27me3 levels and reactivated the KRT13 transcription in the SAS cells (Figures 5 and 6). KRT13 is also expressed in the differentiated epithelial cells of the suprabasal layer [11–13], while Ezh2 is expressed in the basal layer of stratified squamous epithelia [28]. In addition to the loss of KRT13, the overexpression of Ezh2 has been reported to correlate with malignant potential and poor prognosis in human OSCCs [6]. Our data suggest that KRT13 is one of the PRC2-repressed target genes in OSCC cells and that the reciprocal expression of KRT13 and PRC2 serves as a useful biomarker for phenotypic characterization of OSCC cells.

## Conclusions

In summary, our study highlights the epigenetic mechanisms of KRT13 silencing in OSCC cells. We identified epigenetic alterations in the KRT13 promoter in OSCC cells and showed that the pharmacological inhibition of PRC2 restored KRT13 expression. To our knowledge, this is the first report on PRC2-mediated epigenetic silencing of KRT13. Our findings may be useful for the development of diagnostic markers and novel therapeutic approaches against OSCCs.

## Electronic supplementary material

Additional file 1: Figure S1: Cytoplasmic expression of KRT13 protein in the HaCaT cells. HaCaT cells were fixed and probed with anti-keratin 13 (EPR3671; Abcam; dilution, 1:100) or normal rabbit IgG (#2729; Cell Signaling Technology; dilution, 1:100), followed by anti-rabbit IgG antibody conjugated with Alexa Fluor 488. Expression of KRT13 protein (green) was analyzed using a confocal microscope (LSM710; Carl Zeiss MicroImaging GmbH, Jena, Germany), and nuclei were visualized with DAPI staining (blue). (TIFF 735 KB)

Additional file 2: Figure S2: Methylation of H3K9 and reduced RNAP II occupancy at the KRT13 promoter in the SAS cells. (A) Methylation of H3K9 were analyzed by ChIP assay using anti-dimethyl histone H3 (Lys9) (#4658; Cell Signaling Technology), anti-trimethyl histone H3 (Lys9) (MA308B; MAB Institute Inc.). Fold enrichment of histone H3K9me2 and K9me3 were normalized to total histone H3 and calculated relative to that of the HaCaT cells. The means ± SEM for each group (n = 3) are shown. Statistical analysis was performed using Student’s *t*-test. *p < 0.05, **p < 0.01. (B) RNA polymerase II (RNAP II) occupancy at the KRT13 promoter was analyzed by ChIP assay using anti-RNAP II (clone 4H8; Active Motif, Carlsbad, CA). Fold enrichment of RNAP II was normalized to negative control IgG and calculated relative to that of the HaCaT cells. The means ± SEM for each group (n = 3) are shown. Statistical analysis was performed using Student’s *t*-test. *p < 0.05. (TIFF 133 KB)
