# Supplementary figures and images for: Epigenetic alterations of the keratin 13 gene in oral squamous cell carcinoma
Source: BMC Cancer. 2014 Dec 20;14:988. doi: 10.1186/1471-2407-14-988 (PMC4364656; doi:10.1186/1471-2407-14-988)

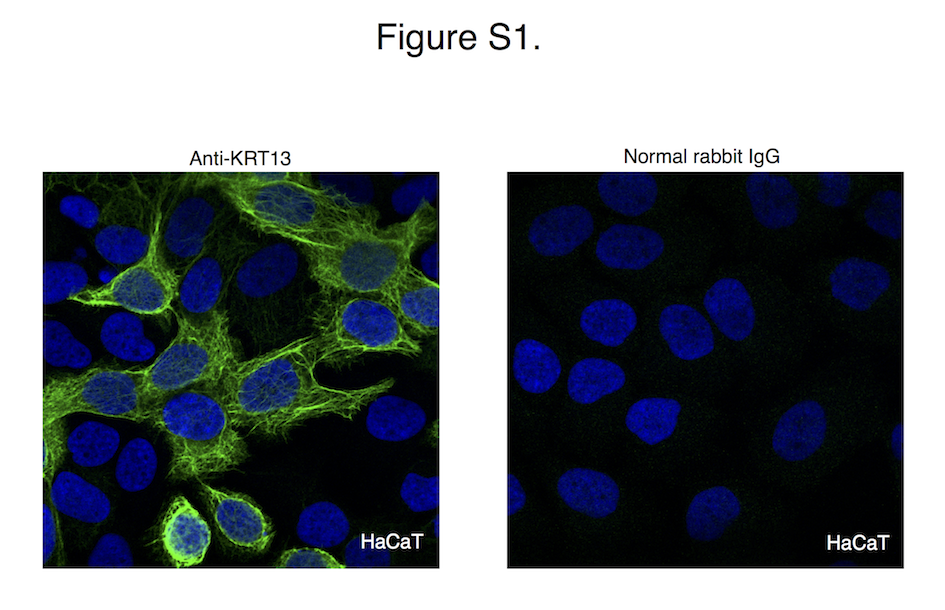

Supplement: Supplementary file 1 — Additional file 1: Figure S1: Cytoplasmic expression of KRT13 protein in the HaCaT cells. HaCaT cells were fixed and probed with anti-keratin 13 (EPR3671; Abcam; dilution, 1:100) or normal rabbit IgG (#2729; Cell Signaling Technology; dilution, 1:100), followed by anti-rabbit IgG antibody conjugated with Alexa Fluor 488. Expression of KRT13 protein (green) was analyzed using a confocal microscope (LSM710; Carl Zeiss MicroImaging GmbH, Jena, Germany), and nuclei were visualized with DAPI staining (blue). (TIFF 735 KB) [file 12885_2014_5157_MOESM1_ESM.tiff]

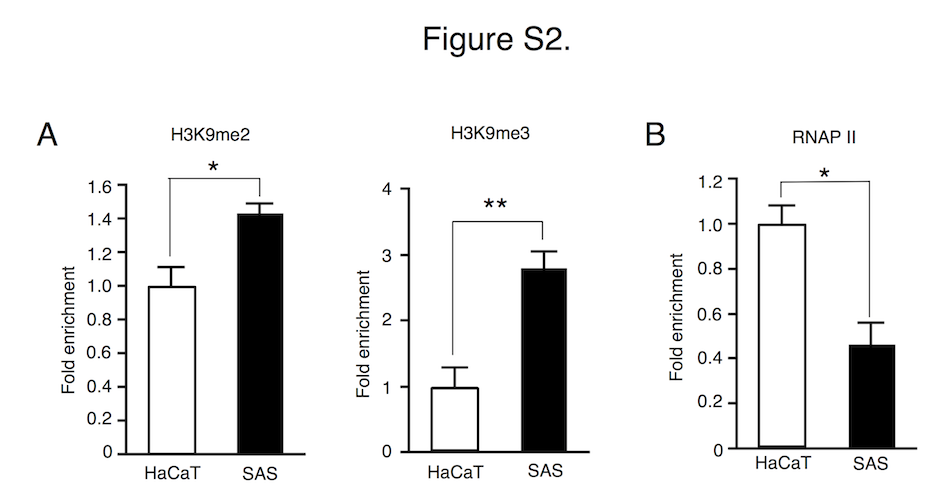

Supplement: Supplementary file 2 — Additional file 2: Figure S2: Methylation of H3K9 and reduced RNAP II occupancy at the KRT13 promoter in the SAS cells. (A) Methylation of H3K9 were analyzed by ChIP assay using anti-dimethyl histone H3 (Lys9) (#4658; Cell Signaling Technology), anti-trimethyl histone H3 (Lys9) (MA308B; MAB Institute Inc.). Fold enrichment of histone H3K9me2 and K9me3 were normalized to total histone H3 and calculated relative to that of the HaCaT cells. The means ± SEM for each group (n = 3) are shown. Statistical analysis was performed using Student’s t-test. *p < 0.05, **p < 0.01. (B) RNA polymerase II (RNAP II) occupancy at the KRT13 promoter was analyzed by ChIP assay using anti-RNAP II (clone 4H8; Active Motif, Carlsbad, CA). Fold enrichment of RNAP II was normalized to negative control IgG and calculated relative to that of the HaCaT cells. The means ± SEM for each group (n = 3) are shown. Statistical analysis was performed using Student’s t-test. *p < 0.05. (TIFF 133 KB) [file 12885_2014_5157_MOESM2_ESM.tiff]
